# Resolution-Associated Lactoferrin Peptides Limit LPS Signaling and Cytokine Secretion from Human Macrophages

**DOI:** 10.3390/ijms21145166

**Published:** 2020-07-21

**Authors:** Aviv Lutaty, Soaad Soboh, Sagie Schif-Zuck, Amiram Ariel

**Affiliations:** The Laboratory for Molecular Pathways in the Resolution of Inflammation, The Department of Human Biology, University of Haifa, Haifa 3498838, Israel; aviv_lut@walla.co.il (A.L.); soaadaboelhija@gmail.com (S.S.); z.sagie@gmail.com (S.S.-Z.)

**Keywords:** resolution of inflammation, lactoferrin, macrophages, LPS, cytokines

## Abstract

The neutrophil granule protein lactoferrin is cleaved and accumulates in efferocytic macrophages as inflammation is resolved. Two peptides present within a resolution-associated 17 kDa fragment of lactoferrin promote the termination of inflammation in vivo by enhancing murine macrophage reprogramming. Here, we report that these two bioactive tripeptides, phenylalanine-lysine-aspartic acid and phenylalanine-lysine-glutamic acid (FKD and FKE, respectively), inhibit ERK and cJun activation following human macrophage exposure to LPS. In addition, these peptides at low concentrations (1–10 μM) modulate human macrophage reprogramming to an anti-inflammatory/pro-resolving phenotype. This was reflected by inhibition of LPS-induced TNF-α and IL-6 secretion and increased IL-10 levels. Moreover, we found naturally occurring FKE analogs (FKECH and FKECHLA) can recapitulate the activity of the short peptide in regulating macrophage cytokine secretion, whereas a reversed EKF peptide was inert in this respect. Curiously, FKD and FKE also regulated cytokine production by bone marrow-derived mouse macrophages, but in a very different fashion than their effect on human macrophages. Thus, lactoferrin peptides limit pro-inflammatory signaling and cytokine production by LPS-activated human macrophages and thereby enhance the resolution of inflammation.

## 1. Introduction

The engulfment of apoptotic polymorphonuclear cells (PMN) by macrophages during the resolution of inflammation is considered to be a hallmark and a major fate-determining event for these cells [1,2,3,4]. The phagocytosis of apoptotic cells and phagolysosome maturation reportedly result in the degradation of the apoptotic cell content [5]. However, neutrophil-derived defensins are preserved and transferred from engulfed apoptotic PMN to the phagolysosomes in the engulfing macrophage and consequently limit intracellular growth of *M. tuberculosis* [6].

Lactoferrin (Lf), an iron-binding glycoprotein from the transferrin family with a molecular weight of 78 kDa, is found in various body fluids like milk, colostrum, saliva, tears, and mucus secretions, as well as in neutrophil secondary granules [7]. Neutrophil degranulation is the main source of Lf in blood [8]. Lf is endowed with a plethora of biological functions, including anti-microbial, anti-viral, anti-parasite, anti-inflammatory and anti-cancer activities [9]. Lf is an important pro-resolving mediator when it is released from apoptotic neutrophils i.e., it blocks the directed migration of neutrophils and eosinophils [10,11]. Several peptides within Lf have previously been shown to have distinct bioactivities that in some cases differ from the parent protein [7,12,13,14]. 

Recent findings indicate a 17 kDa fragment of Lf is released to interstitial spaces during the resolution phase of murine peritonitis and bovine mastitis, but not during the onset of disease [15]. This fragment is generated by neutrophil serine proteases prior to PMN efferocytosis by resolution phase macrophages and contains two tripeptides, phenylalanine (Phe)—lysine (Lys)—aspartic acid (Asp) and phenylalanine (Phe)—lysine (Lys)—glutamic acid (Glu) (FKD and FKE, respectively), which harbor important pro-resolving properties in vivo [15]. Both peptides enhanced the generation of pro-resolving CD11b^low^ macrophages [16,17] in vivo. The FKE peptide also enhanced macrophage reprogramming by promoting the secretion of an anti-inflammatory/pro-resolving cytokine profile, while the FKD peptide had an opposite effect in vivo [15]. The later finding is consistent with previous reports on the activating nature of FKD-containing peptides in bovine and human settings [13,14]. 

Here, we report that the FKE and FKD peptides regulate similar signal transduction modules in human macrophages, such as cJun, ERK and p38MAPK, but with different kinetics. Moreover, both Lf peptides (LfP) limited ERK and cJun activation by LPS in these cells but did not affect p38MAPK activation. In addition, FKE limited LPS-induced inflammatory cytokines and TGF-β, while both peptides enhanced IL-10 secretion by human macrophages. The actions of FKE were mimicked, to some extent, by Lf-derived extended analogs of this peptide, as well as the murine homologue, YKE, but not by the reversed peptide EKF. Thus, LfP act on human macrophages to inhibit pro-inflammatory signaling and cytokine secretion and promote reprogramming.

## 2. Results

### 2.1. The Lf-Derived Peptides FKD and FKE Modulate ERK1/2, P38MAPK and cJun Signaling in Human Macrophages

**Figure 1:**
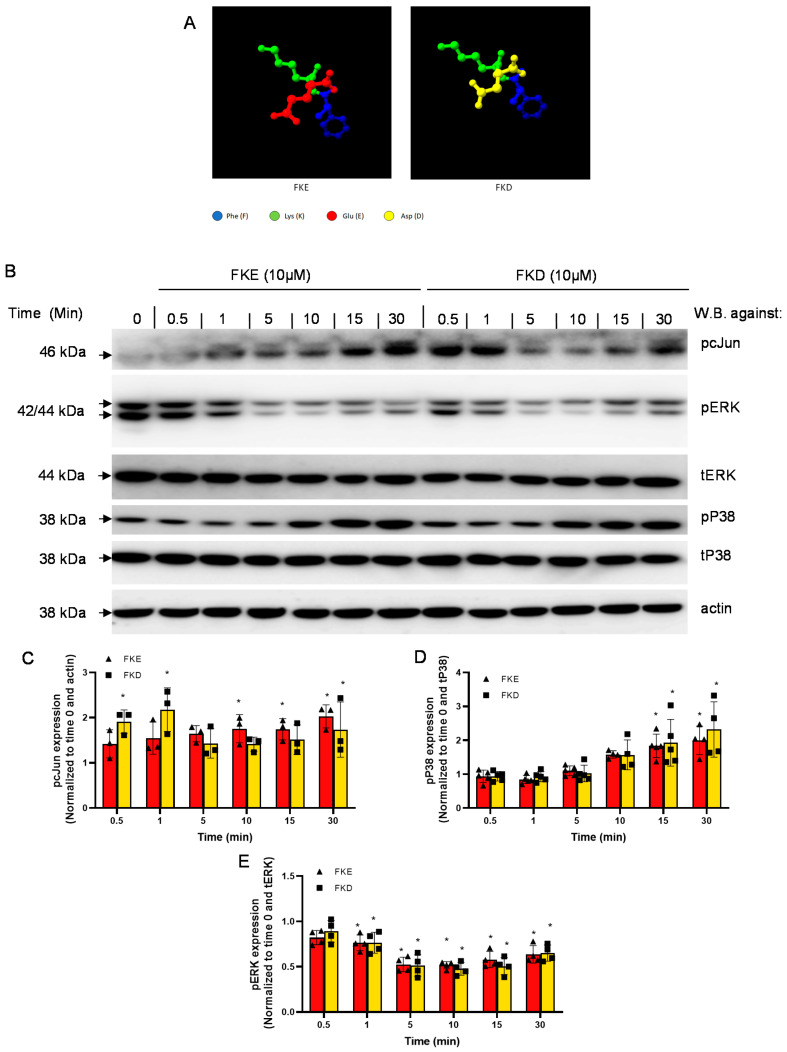
LfP activate P38MAPK and cJun and inhibit ERK in U937-derived human macrophages. U937-derived macrophages were treated with vehicle, FKE or FKD (10 µM; structure depicted in (**A**) for the indicated periods, then the cells were harvested and protein extracts from the cells were run by 10% SDS-PAGE, and blotted for total or phospho-ERK1/2, P38MAPK, and cJun, as well as actin (**B**) using the appropriate primary and secondary antibodies. Densitometric analysis for phospho-cJun (**C**), phospho-P38MAPK (**D**) and phospho-ERK1/2 (**E**) are depicted following normalization to actin, total P38MAPK and ERK, respectively, and to untreated cells (Time 0). Results are representative (**B**) and means ± SD from 3–4 experiments. * *p* < 0.05 compared to untreated cells (Time 0) (ANOVA).

LfP were found to exert pro-resolving and reprogramming actions on murine macrophages in vivo [15]. Therefore, we aimed to determine which signaling pathways are regulated by these peptides in human macrophages and whether the subtle difference in their structure (Figure 1A) leads to differences in their intracellular activity. To this end, we examined three major signal transduction pathways previously found to be involved in cytokine production, particularly following LPS exposure: ERK1/2, P38MAPK and cJun [18,19]. We initially determined by western blotting whether these molecules are activated (by undergoing phosphorylation) in U937-derived macrophages following treatment with organically-synthesized FKD or FKE. Our results (Figure 1B–E) show that FKE and FKD induced the phosphorylation of cJun (Figure 1C) and P38MAPK (Figure 1D), while reducing the phosphorylation of ERK1/2 (Figure 1E) in a temporal manner. The activation of cJun and inhibition of ERK took place earlier (0.5–1 min post exposure) and FKE trailed FKD in the starting point. The activation of P38MAPK, on the other hand, occurred 10 min post exposure to the peptides and was similar in its kinetics when comparing both peptides. Importantly, the extended FKECHLA peptide showed a similar response to the FKE peptide albeit with much higher activation of cJun and P38MAPK (data not shown). 

**Figure 2:**
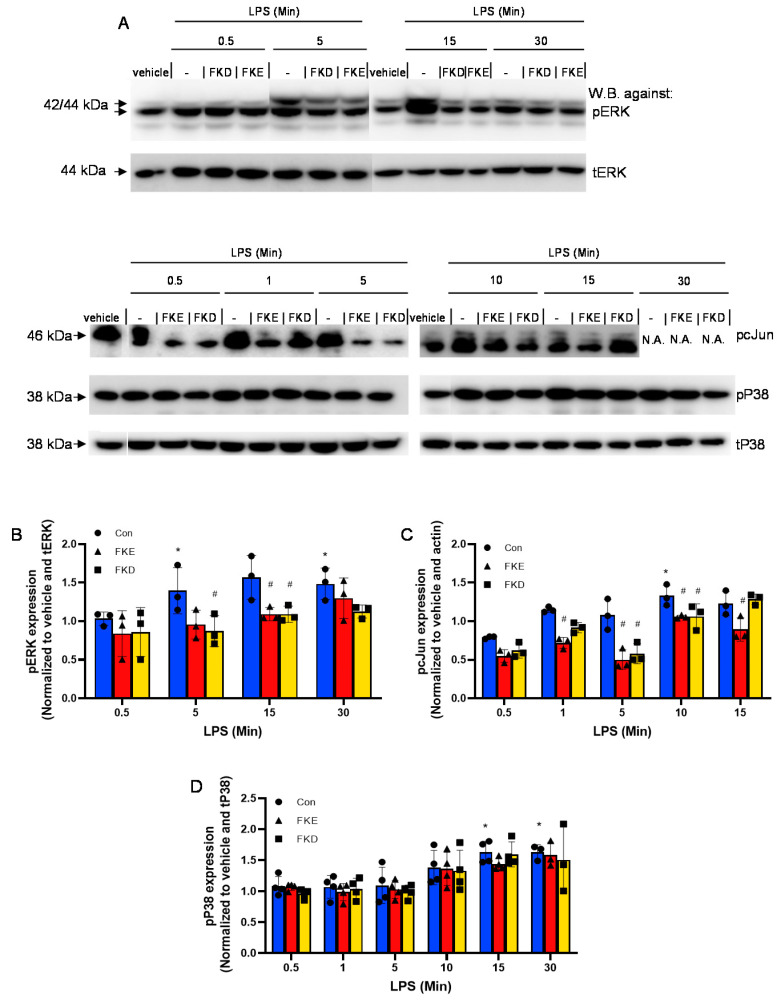
LfP inhibit LPS-induced ERK and cJun activation in human macrophages. U937-derived macrophages were treated with FKE or FKD (10 µM) for 60 min, and then supplemented with LPS (10 ng/mL). After additional 0.5–30 min the cells were harvested and protein extracts from the cells were run by 10% SDS-PAGE, and blotted for total or phospho-ERK1/2, P38MAPK, and cJun using the appropriate primary and secondary antibodies. Results are representative from three experiments (**A**). Time points that showed unequal amounts of total proteins were excised from the presented image. Densitometric analysis for phospho-ERK1/2 (**B**), phospho-cJun (**C**) and phospho-P38MAPK (**D**) are depicted following normalization to total ERK, actin, and total P38MAPK, respectively, and to controls/vehicle-treated cells (Con). Results are representative (**A**) and means ± SD (**B**–**D**) from 3–4 experiments. * *p* < 0.05 compared to untreated cells, ^#^ *p* < 0.05 compared to LPS-treated cells at the same time (ANOVA).

Next, we examined whether LfP affected LPS-induced phosphorylation of ERK, P38MAPK, or cJun. Our results (Figure 2A) show that both FKE and FKD inhibited the transient upregulation in phospho-ERK induced by LPS, especially at the 15 min time point (Figure 2B). In addition, they reduced cJun phosphorylation during its induction by LPS, with better inhibition obtained by FKE (at 1 and 15 min) in comparison to FKD (Figure 2C). No significant inhibition of LPS-induced P38MAPK phosphorylation was detected by LfP (Figure 2D). Thus, our results indicate FKE and FKD peptides differentially regulate ERK, P38MAPK and cJun activation when acting alone or in concert with LPS signaling.

### 2.2. LfP Regulate Cytokine Production from Human Macrophages

**Figure 3:**
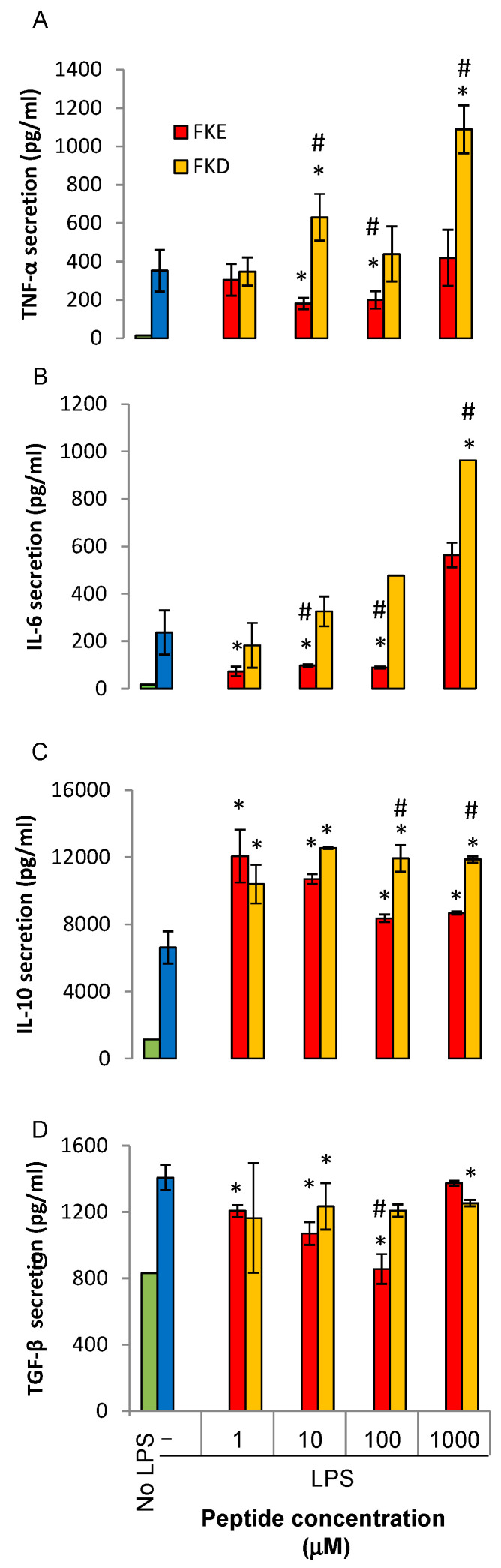
Lf-derived peptides regulate LPS-induced cytokine secretion from human macrophages. U937-derived macrophages were treated with FKE or FKD peptides (0.1–1000 µM) for 24 h and then treated with LPS (100 ng/mL, 24 h). Next, culture media was collected and its cytokine content was determined by standard ELISA for TNF-α (**A**), IL-6 (**B**), IL-10 (**C**) or TGF-β (**D**). Results are representative from five experiments with biological and analytical duplicates for each treatment and are presented as mean ± SD. * and ^#^ indicate *p* < 0.05 compared to treatment with LPS alone or with other peptide, respectively. All treatments were statistically different from untreated cells, except cells treated by LPS and 100 μM FKE in (D) (ANOVA).

**Figure 4:**
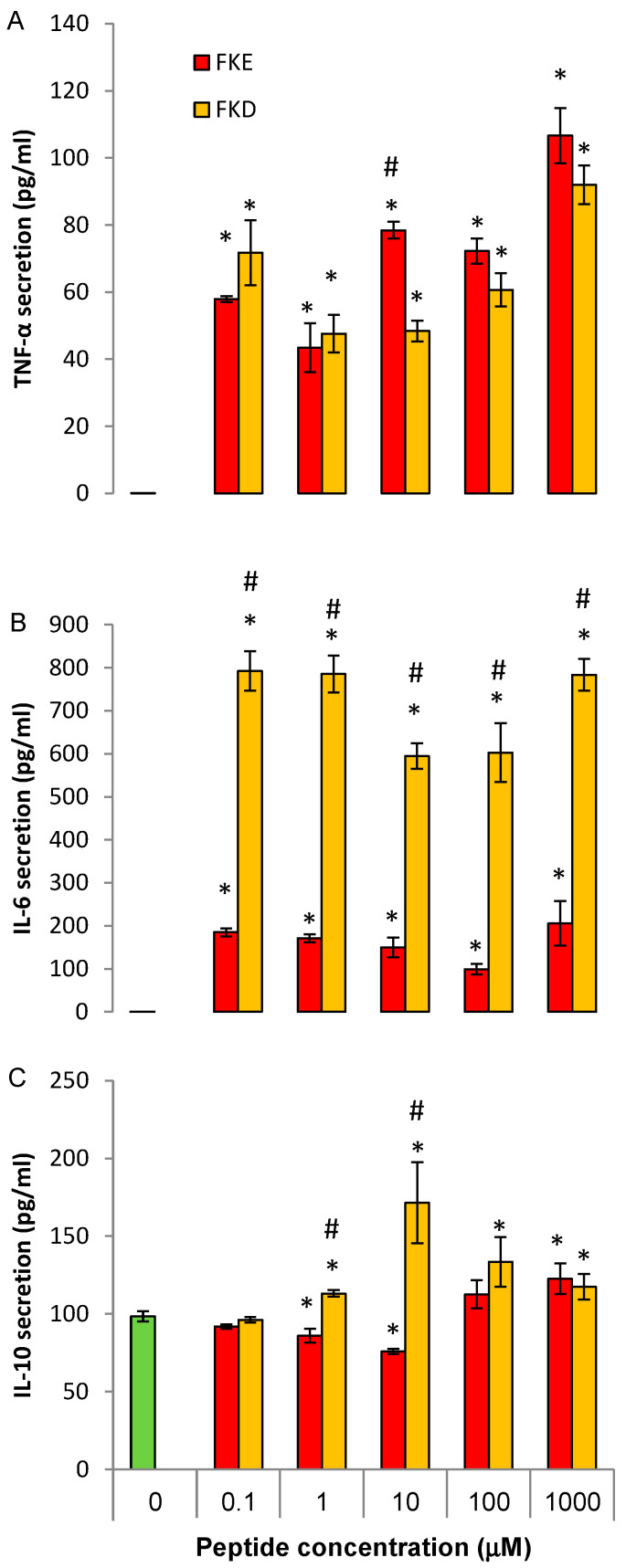
Lf-derived peptides regulate cytokine secretion from human macrophages. U937-derived macrophages were treated with FKE or FKD peptides (0.1–1000 µM) for 24 h. Then, culture media was collected and its cytokine content was determined by standard ELISA for TNF-α (**A**), IL-6 (**B**), or IL-10 (**C**). Results are representative from five experiments and presented as mean ± SD with biological and analytical duplicates for each treatment. * and ^#^ indicate *p* < 0.05 compared to treatment with vehicle or with other peptide, respectively.

We have previously shown that LfP modulate cytokine production by murine macrophages with FKE promoting an anti-inflammatory profile, while FKD promoted a pro-inflammatory one [15]. Therefore, we determined whether these peptides exert anti-inflammatory and pro-resolving actions and whether the FKD and FKE peptides differ in this respect. To this end, we treated U937-derived human macrophages with increasing concentrations of FKE and FKD tripeptides, and consequently activated them with LPS or left them untreated and measured the secretion of TNF-α, IL-6, IL-10, and TGF-β after an additional 24 h. Our results, shown in Figure 3, indicate FKD enhanced the LPS-induced secretion of both pro-inflammatory cytokines (IL-6 and TNF-α) whereas FKE inhibited the secretion of these cytokines at 1–100 μM (Figure 3A,B, respectively). FKE also enhanced IL-10 secretion at 1 µM but this effect was reduced at higher concentrations. FKD, on the other hand showed saturated enhancement starting at 10 μM. With regards to TGF-β, FKE (at 100 μM) completely inhibited its LPS-induced secretion, whereas FKD inhibited it by 33% at 10–100 μM. Notably, both peptides inhibited TNF-α secretion at 24 h in the absence of LPS (data not shown, *n* = 2), but later increased the residual secretion at 48 h (Figure 4A). A similar effect was found with regards to IL-6 (Figure 4B) albeit the FKD peptide was much more potent than its FKE counterpart. Notably, only the FKD peptide increased secretion of IL-10 (by 70% at 10 μM) in the absence of LPS, whereas FKE inhibited it by 25% at the same concentration (Figure 4C). Thus, our results indicate FKE, but not FKD, inhibited LPS-induced inflammatory cytokine secretion from human macrophages, whereas both peptides upregulated the secretion of IL-10 and inhibited TGF-β under this setting.

### 2.3. FKE Analogs Mimic its Regulation of Cytokine Production from Human Macrophages

**Figure 5:**
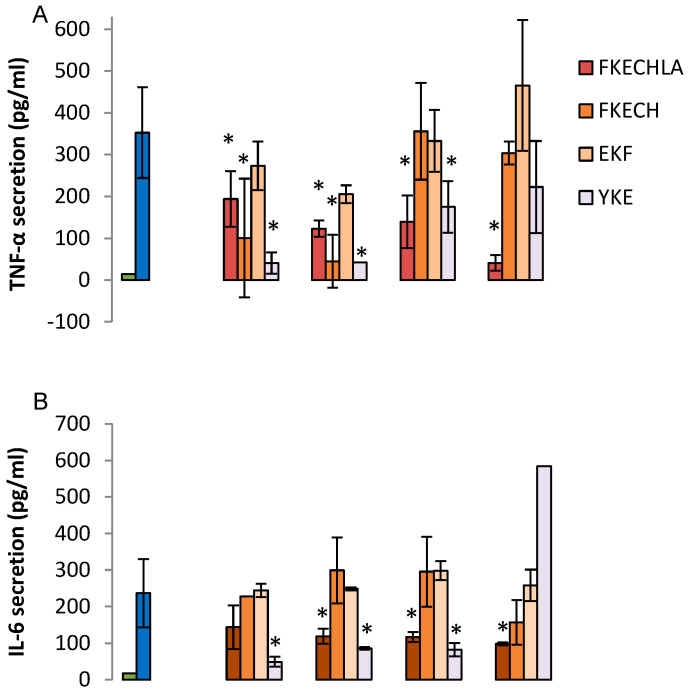

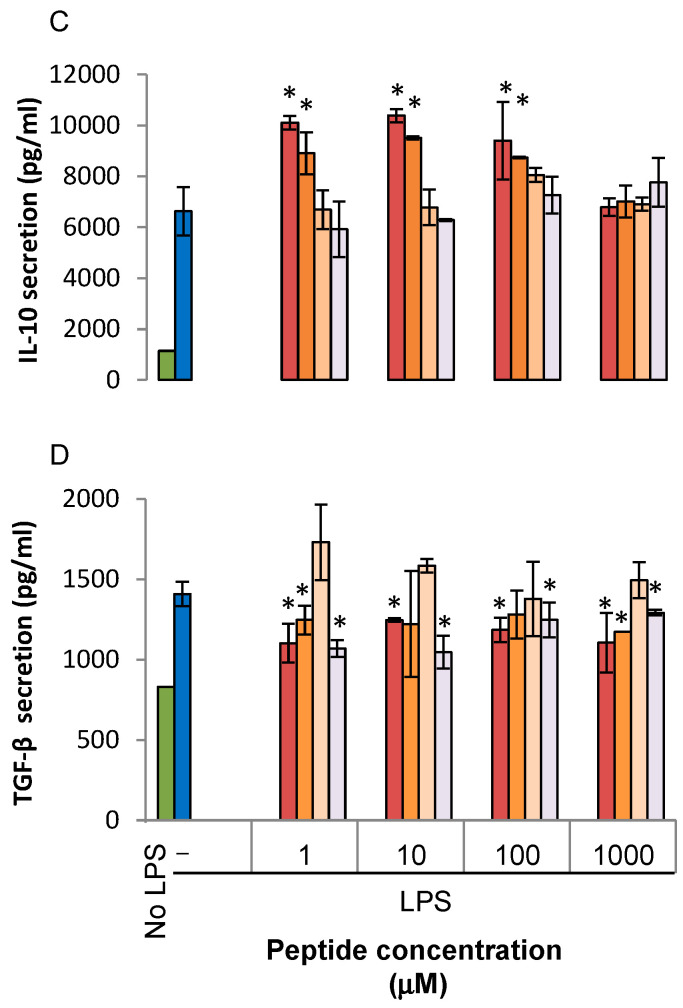
Regulation of LPS-induced cytokine secretion by LfP analogs. U937-derived macrophages were treated with FKECHLA, FKECH, EKF or YKE peptides (1–1000 µM) for 24 h and then treated with LPS (100 ng/mL, 24 h). Culture media was collected and its cytokine content was determined by standard ELISA for TNF-α (**A**), IL-6 (**B**), IL-10 (**C**) or TGF-β (**D**). Results are representative from three experiments and presented as mean ± SD with biological and analytical duplicates for each treatment. * indicate *p* < 0.05 compared to treatment with LPS alone. All treatments were statistically different from untreated cells, except cells treated by LPS and 1 or 10 μM FKECH in (**A**) (ANOVA).

Since the FKE peptide was shown to be part of a larger peptide generated by serine proteases (FKECHLA) we determined which amino acids in the larger peptide are necessary/sufficient to reproduce the regulatory actions of FKE. Moreover, the FKE analog in murine Lf, YKE [11] was also of interest as well as the control reversed peptide (EKF). Therefore, we determined the regulatory properties of FKECH, FKECHLA, YKE, and EKF peptides on LPS-stimulated cytokine secretion from human macrophages. Our results (Figure 5A–D) show that FKECH peptide reproduced the actions of FKE in inhibiting TNF-α, stimulating IL-10, and inhibiting TGF-β secretion, but was ineffective in reducing IL-6 below 1000 µM.

Notably, the FKECHLA peptide showed lower inhibition of TNF-α and IL-6 secretion at low concentrations (1–100 μM) but improved this inhibition at 1 mM, whereas FKE was stimulatory at this concentration (Figure 3A,B). With regards to IL-10 and TGF-β, FKECHLA essentially recapitulated the effects of FKE. Interestingly, the YKE peptide recapitulated the actions of FKE in regulating the secretion of TNF-α, IL-6, and TGF-β, but was totally inactive in regulating IL-10. As expected, the EKF peptide was devoid of regulatory properties on any of the cytokines. Therefore, specific modifications of the FKE sequence seem to modulate specific properties of this peptide while preserving its basic activities. Notably, the FKECHLA peptide maintained its inhibitory nature through all concentrations examined; hence suggesting it is a better pro-resolving agent than the minimal FKE tripeptide. Altogether, these results indicate FKE, its physiological counterpart, its murine analog, and to a certain degree FKD, regulate cytokine secretion in LPS-activated human macrophages, thereby promoting their reprogramming.

### 2.4. LfP Regulate Cytokine Production by Murine Bone Marrow Macrophages

**Figure 6:**
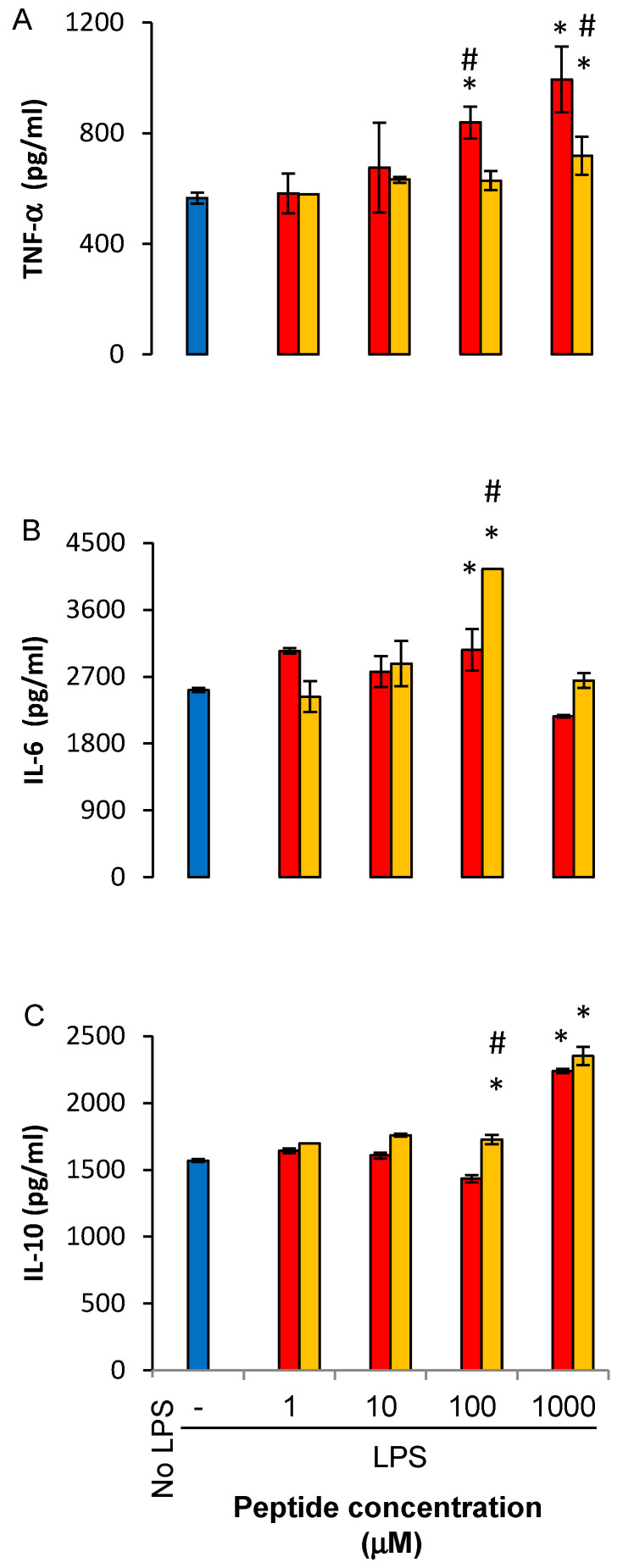
LfP regulate cytokine secretion from murine bone marrow macrophages. Bone marrow macrophages from unchallenged mice were treated with FKE or FKD peptides (1–1000 µM) for 24 h, followed by LPS exposure for additional 24 h. Next, culture media was collected and its cytokine content was determined by standard ELISA for TNF-α (**A**), IL-6 (**B**), or IL-10 (**C**). Results are representative from two experiments (*n* = 4 mice for each experiment) and presented as mean ± SD with biological and analytical duplicates for each treatment. * and ^#^ indicate *p* < 0.05 compared to treatment with LPS alone or with other peptide, respectively. All treatments were statistically different from untreated cells (ANOVA).

Treatment with LfP in vivo regulates cytokine production by LPS-stimulated resolution phase macrophages [11]. To determine whether LfP have direct effect on murine macrophages, we isolated F4/80^+^ bone marrow macrophages (BMM) from unchallenged mice and treated them with LfP for 24 h, followed by LPS exposure. Our results (Figure 6) show LfP affected LPS-induced cytokine secretion from BMM but in a very different manner than their regulation of human macrophages. FKE, but not FKD, increased TNF-α secretion in a concentration-dependent manner that maximized at 1000 μM (Figure 6A). IL-6 secretion was increased by FKD, but not FKE, and only at 100 μM (Figure 6B). IL-10 secretion was increased by both peptides, only at 1000 µM (Figure 6C). Interestingly, IL-12 production by BMM was significantly inhibited at all concentrations by FKD and to a much lower extent by FKE (data not shown; *n* = 2). Thus, the anti-inflammatory activity of LfP is significantly hindered in BMM, while the upregulation of IL-10 takes place only at high concentrations.

## 3. Discussion

Previous reports have indicated that lactoferrin is cleaved to several short peptides that act on various cell types from murine, bovine and human origin [13,14,15,20]. During bacterial infection, a Lf fragment with 22 kDa was previously described in bovine and human samples [13,14]. This fragment contained four peptides generated by the serine proteases elastase and proteinase 3. One of these peptides with the sequences PGQRDLLFKDSAL/SGQKDLLFKDSAI in bovine and human Lf, respectively, induced cytokine and chemokine secretion in epithelial cells. Another peptide present in human Lf, FKDCHLA, induced inflammatory cytokine secretion, while its bovine homologue FKECHLA was inactive. It seems that the FKD tripeptide present in the first three peptides might account for their activity while the replacement of aspartic acid to glutamic acid (Figure 1A,D,E, respectively) results in the abortion of the stimulatory activity. Along these lines, we have recently reported that FKE peptides promote murine resolution phase macrophage reprograming [15], while FKD was unable to do so. Reprogramming of macrophages resulted in increased uptake of apoptotic targets alongside reduced secretion of TNF-α and IL-6, while IL-10 secretion was enhanced following LPS exposure. To extend these findings to the human setting and examine the signaling pathways involved in LfP activity, we examined whether FKE, FKD or analogs of FKE modulated intracellular kinases and transcription factors involved in cytokine production. We examined three major signal transduction pathways previously found to be involved in macrophage activation, especially following exposure to LPS: ERK1/2, P38MAPK and cJun [18,19]. Some 5–30 min after treatment, LfP induced a mild phosphorylation of cJun and P38MAPK, while they inhibited ERK phosphorylation (Figure 1). FKE and FKD differed in the magnitude and kinetics of induction of cJun phosphorylation while they showed very similar patterns in terms of pERK1/2 inhibition and P38MAPK activation. This potentially accounts for the differences observed in the inhibition of cytokine secretion by these peptides upon LPS stimulation (Figure 3). Notably, both peptides inhibited ERK1/2 phosphorylation induced by LPS. Moreover, both peptides inhibited LPS-induced phosphorylation of cJun, with greater inhibition by FKE at 1 and 15 min following treatment (Figure 2). Neither FKE nor FKD inhibited LPS-induced P38MAPK phosphorylation indicating selective inhibition of distinct signaling pathways rather than modulation of TLR4 expression. 

We also determined whether LfP and FKE analogs shift cytokine secretion by human macrophages toward an anti-inflammatory profile. We found (Figure 3) that at low concentrations (1–100 μM) both peptides enhanced IL-10 secretion induced by LPS. The FKE peptide also inhibited TNF-α and IL-6 secretion at these concentrations, indicating it promoted macrophage reprogramming rather than non-selective LPS responsiveness due to a reduction in TLR4 expression. FKD, on the other hand, did not affect LPS-induced TNF-α and IL-6 secretion at low concentrations but significantly enhanced it at 1000 μM. 1000 μM FKE displayed a stimulatory effect as well. However, this concentration may not be reached in vivo. Importantly, the LPS-induced secretion of TGF-β, a key effector cytokine involved in the resolution of inflammation as well as in tissue fibrosis [21] was completely inhibited by FKE, and to a lower extent by FKD. The parent Lf-derived FKECHLA peptide was able to reproduce the actions of FKE (with respect to TNF-α and IL-6) albeit at higher concentrations and without reaching a stimulatory concentration at the range examined. Thus, FKECHLA seems to exert lower activity than FKE. Notably, the mouse homologue of FKE, YKE, reproduced FKE’s inhibition of TNF-α, IL-6, and TGF-β, but did not stimulate IL-10 secretion. These findings suggest that despite the similarity in structure of FKE and YKE peptides, the signaling pathway that activates IL-10 production is not engaged following the phenylalanine to tyrosine (F to Y, respectively) replacement. Together, our findings suggest that peptides present within the resolution-associated Lf fragment can modulate human macrophage reprogramming toward a pro-resolving phenotype at low, inhibitory, concentrations through the inhibition of LPS-induced activation of ERK and cJun, but not P38MAPK.

Our findings are in agreement with previous publications that indicated phagocytosis of apoptotic cells by macrophages results in a similar modulation of phosphorylation patterns: activation of both P38MAPK and Jun kinase/cJun and inhibition of ERK that is essential for the inhibition of inflammatory cytokine secretion upon LPS activation [22,23]. Putting these findings in context with our current results suggests that LfP are, at least partially, mimicking the effect of phagocytosis of apoptotic cells by macrophages. As a result both LfP and apoptotic cells induce macrophage immune-silencing/reprogramming manifested by downregulation of pro-inflammatory cytokine secretion while the anti-inflammatory IL-10 is upregulated [24,25]. Although their complete mechanism of action requires further research, our current results imply that LfP act through a membrane receptor-mediated intracellular signaling cascade. This is suggested by their bioactivity at low concentrations and the induction of P38MAPK and cJun phosphorylation in kinetics resembling TLR-binding molecules, like LPS, and cytokines. Thus, we suggest LfP bind yet unknown surface receptor(s) and consequently activate an intracellular signal transduction pathway that stimulates neutrophil NETosis [15] but limits macrophage activation by LPS, essentially acting as partial agonists. Notably, LfP did not significantly reduce inflammatory cytokine production by murine BMM at low concentrations, likely due to low expression of the putative receptor in these cells. Nevertheless, lactoferrin peptides seem to be a promising new approach to treat inflammatory and fibrotic human diseases.

## 4. Materials and Methods 

### 4.1. Reagents

The following reagents were purchased as detailed: acrylamide/bisacrylamide, LPS (from *E. coli,* clone 055:B5), TEMED, and Tween-20 from Sigma-Aldrich Israel Ltd. (Rehovot, Israel); anti-rabbit horseradish peroxidase-conjugated IgG from Jackson Immuno Research Laboratories (West Grove, PA, USA); EZ-ECL, fetal calf serum, L-glutamine, penicillin-streptomycin, RPMI 1640 and trypan blue from Biological Industries (Kibbutz Beit Haemek, Israel); ELISA kits for human IL-10 and TGF-β from BioLegend (San Diego, CA, USA); ELISA kits for human TNF-α and IL-6 from BD Bioscience (San Jose, CA, USA); and protease inhibitors cocktail was purchase from Roche (Basel, Switzerland). FKD, FKE, FKECH, FKECHLA, YKE, and EKF peptides were synthesized by GL Biochem (Shanghai) Ltd. (Shanghai, China) with purity typically over 90%. 

### 4.2. Regulation of Signal Transduction

U937-derived human macrophages were treated with FKE or FKD for 0.5–30 min. Then, the cells were washed with cold PBS and put on ice. Next, the cells were lysed with RIPA lysis buffer (containing 10 µM PMSF, 10 mM sodium orthovanadate and 1:25 diluted protease inhibitors cocktail, 10 min on ice). Then, lysates were collected, added with sample buffer (1:5) and boiled. Equal amount of protein were run by 10% SDS-PAGE and blotted with the following primary antibodies: rabbit anti-human phospho- or total-P38MAPK, rabbit anti-human phospho- or total-ERK or rabbit anti-human phospho-cJun (all from Cell Signaling Technology, Vancouver, BC, Canada) and the appropriate secondary antibodies. Membranes were developed with EZ-ECL detection kit (Biological Industries) and analyzed using a Luminescent Image Analyzer LAS-4000 (Fujifilm Corporation, Tokyo, Japan) and Image Reader LAS-4000 software (Fujifilm Corporation).

### 4.3. Regulation of Cytokine Secretion from Murine and Human Macrophages by LfP

Bone marrow (BM) macrophages were isolated following sterilization of the abdomen and hind legs of unchallenged mice with 70% ethanol. Pelvic and femoral bones were dissected and excess muscle from legs was removed. Each bone end was cut off and BM was flushed with 1 mL of PBS until bone cavity appeared white. Then, cells were centrifuged at 1200 rpm for 7 min, and the supernatant was discarded. Cell pellet was resuspended in 1% BSA in PBS and macrophages thereof were isolated using EasySep PE selection magnetic beads directed against F4/80, according to the manufacturer’s instructions (StemCell Technologies, Vancouver, BC, Canada). The protocol was approved by the ethics committee for animal experimentation of the University of Haifa (authorization no. 246/14). Human monocytic U937 cells were differentiated to macrophages by exposure to 50 ng/mL of phorbol myristate acetate (PMA) for 48 h at 37 °C, 5% CO_2_ humidified atmosphere. After differentiation, macrophages were washed twice with culture media (RPMI 1640 containing 10% fetal bovine serum (FBS), 100 units/mL penicillin, 100 μg/mL streptomycin and 0.01% β-mercaptoethanol), and then incubated with the various LfP or their analogs (FKE, FKD, FKECH, FKECHLA, YKE, or EKF) at 0.1, 1, 10, 100 and 1000 µM or culture media as control for 24 h. Then, supernatants were collected and the cells were further incubated with culture media or with LPS (100 ng/mL) for additional 24 h. Then, culture supernatants were collected and evaluated for TNF-α, IL-6, IL-10, and TGF-β, using standard ELISA.

### 4.4. Data Analysis

Experimental data were analyzed by Student’s t-test or ANOVA (Tukey’s HSD) with *p* values ≤ 0.05 designated as statistically significant. Results are presented as means ± standard deviation.

## 5. Patents

A patent application in which Amiram Ariel, Aviv Lutaty and Sagie Schif-Zuck are inventors has been issued for the data presented in the manuscript: Synthetic anti-inflammatory peptides and use thereof (WO 2014174517 A1, April 25th, 2013).

## Abbreviations

BMEC	Bovine mammary gland epithelial cells
ELISA	Enzyme-linked immunosorbent assay
ERK	Extracellular signal-regulated kinase
FCS	Fetal calf serum
IgG	Immunoglobulin G
IL	Interleukin
kDa	kiloDalton
Lf	Lactoferrin
LfP	Lactoferrin peptides
LPS	Lipopolysaccharide
MCP-1	Monocyte chemotactic protein-1
NF-kB	Nuclear factor kappa B
P38MAPK	p38 Mitogen-activated protein kinase
PMA	Phorbol myristate acetate
PMN	Polymorphonuclear cells
PVDF	Polyvinylidene difluoride
RIPA	Radioimmunoprecipitation assay
SDS-	Sodium dodecyl sulfate
PAGE	Polyacrylamide gel electrophoresis
TGF-β	Transforming growth factor-β
TNF-α	Tumor necrosis factor-α
